# The curative effect of traditional Chinese medicine fumigation combined with acupotomy in the treatment of heel pain: A meta-analysis

**DOI:** 10.1097/MD.0000000000043249

**Published:** 2025-08-22

**Authors:** Chen Li, Zhi-Wen Sun, Jia-Xin Ma, Xi Gao, Hong-Jun Lou, Cheng-Yuan Li

**Affiliations:** a Fourth Department of Bone Injury Diseases, First Affiliated Hospital of Heilongjiang University of Chinese Medicine, Harbin, China; b First Department of Digestive Diseases, First Affiliated Hospital of Heilongjiang University of Chinese Medicine, Harbin, China; c School of Graduate, Heilongjiang University of Chinese Medicine, Harbin, China; d Department of Clinical Laboratory, First Affiliated Hospital of Heilongjiang University of Chinese Medicine, Harbin, China.

**Keywords:** acupotomy, GRADE system, heel pain, meta-analysis, traditional Chinese medicine fumigation

## Abstract

**Background:**

To evaluate the clinical therapeutic effect of traditional Chinese medicine (TCM) fumigation combined with acupotomy for treatment of heel pain.

**Methods:**

After a comprehensive search of large databases globally, a meta-analysis was performed on the results of randomized controlled trials conforming to the inclusion criteria using Review Manager 5.4. software, and the quality of the evidence rated using the Grading of Recommendations Assessment, Development and Evaluation profiler 3.2.2 software.

**Results:**

A total of 1095 patients were included in 12 studies, with 548 in the treatment group and 547 in the control group. Heterogeneity test analysis showed no statistical heterogeneity among the 12 studies; therefore, a fixed-effects model combined with effect size analysis was used. The results of the meta-analysis showed that the clinical difference between the experimental and control groups was statistically significant. Grading of Recommendations Assessment, Development and Evaluation system evidence grading showed that the evidence grade of TCM fumigation combined with acupotomy for heel pain was low.

**Conclusions:**

The curative effect of TCM fumigation combined with acupotomy in the treatment of heel pain was better than that of medicine alone. The evidence quality level of this conclusion was not high, and the research conclusion should be treated with caution and further confirmed using large samples and high-quality clinical trials.

## 1. Introduction

Heel pain, an orthopedic disease commonly encountered in clinical practice, has a higher incidence rate in middle-aged and elderly individuals, athletes, those who stand for long periods of time, and obese individuals.^[1]^ The clinical manifestations of this condition primarily include severe pain in the heel, either unilaterally or bilaterally, which can be unbearable. Patients may also experience soreness, swelling, or a severe needling sensation on the sole of the foot. They may be afraid to touch the ground, have difficulty bearing weight, and may require assistance to move, which significantly impacts their daily life.^[2]^ The pathogenesis of heel pain is multifactorial. Current medical research indicates that common causes of heel pain include conditions such as heel osteoarthritis, plantar fasciitis, retrocalcaneal bursitis, calcaneal periostitis, abnormal arch structure development, Achilles tendonitis, and inflammation of the heel fat pad. During walking or standing, the bone spur can rub against surrounding soft tissues, leading to tissue damage and triggering local aseptic inflammation in the heel area. The resulting inflammation and metabolic byproducts can irritate nerve endings in the foot, leading to pain and discomfort. Moreover, the presence of bone spurs can exert pressure on the skin and soft tissues of the foot sole during walking, causing blood pooling in the heel bone and increased intraosseous pressure, contributing to the sensation of pain. Current treatment methods such as oral nonsteroidal anti-inflammatory drugs, local hyperthermia physiotherapy, external application of drugs to promote blood circulation and dredge collaterals, and local injection of prednisolone drugs have limited effectiveness in relieving pain and often lead to disease relapse, leaving patients dissatisfied with the expected results.^[3]^ Acupotomy, a form of closed therapy that uses a miniature surgical instrument composed of a handle, needle body, and blade, has shown promise for relieving heel pain. It works by loosening the adhesion tissue of the heel, modifying the thickness of the plantar fascia, reducing plantar stress, and aiding in restoring the normal strength of the foot. This therapy helps restore the dynamic balance of local tissue with minimal side effects and is a simple procedure.^[4–8]^ Traditional Chinese medicine (TCM) fumigation encompasses medicinal gas baths and Chinese herbal medicine foot soaking, among other treatment forms. However, it is important to note that TCM fumigation and washing combined with acupotomy may not provide a complete solution for all symptoms of heel pain. Nevertheless, several clinical studies have demonstrated the effectiveness of this combined therapy, which integrates traditional Chinese and Western medicine and falls under the category of closed therapy.^[9]^ This approach has been demonstrated to be more effective than conservative therapy in restoring local dynamic balance. Moreover, research suggests that the combination of internal and external therapies yields more significant results than a single therapy. Importantly, this combined therapy is not only effective but also safe and reliable.^[8]^ In recent years, there has been increasing use of TCM fumigation combined with acupotomy for heel pain in clinical settings, with numerous clinical research reports and randomized controlled trials (RCTs) supporting its efficacy. A meta-analysis based on high-quality RCTs serves as a crucial evidence base for clinical practice, medical education, and decision-making. In this study, we employed a meta-analysis method to quantitatively analyze multiple independent clinical studies and used the internationally recognized Grading of Recommendations Assessment, Development and Evaluation (GRADE) system to assess the quality of evidence for outcome indicators. This comprehensive approach allows for an objective evaluation of the effectiveness of TCM fumigation and acupotomy in treating heel pain.^[10]^

## 2. Materials and methods

This meta-analysis was performed according to the PRISMA 2020 guidelines for systematic reviews and meta-analyses.^[11]^

### 2.1. Protocol and registration

This review has been registered in the International Prospective Register of Systematic Reviews (PROSPERO), the registration number is CRD42023434362.

### 2.2. Search strategies

The retrieval process was conducted from January 2000 to July 2023, using a systematic approach. Computer retrieval was the primary method used, supplemented by manual retrieval. The search was performed using various databases, including PubMed, Cochrane Library, Embase, SCOPUS, Web of Science core, Chinese Biomedical Literature Database, China National Knowledge Infrastructure, Wanfang Digital Periodical Group, and the VIP Database. Relevant RCTs meeting the inclusion and exclusion criteria were searched without language restrictions. The literature search focused on keywords, synonyms, substitute words, and disease names related to the treatment of heel pain using TCM fumigation and washing combined with acupotomy. A literature retrieval formula was established to facilitate this search. Two reviewers initially screened the literature by reviewing titles and abstracts and then assessed the full text of potentially eligible trials to determine their inclusion in the meta-analysis. In case of disagreement, a third party was consulted. The reference lists of the identified articles were examined to identify additional relevant studies.

### 2.3. Document inclusion criteria

#### 2.3.1. Research type

(1) RCT clinical research, which covered literature that clearly indicated random grouping by methods such as coin toss, turntable method, or computer programming, or records random grouping but did not specify which method was used. Studies were included regardless of whether they were single-blind or double-blind. (2) The treatment group used TCM fumigation and acupuncture combined with acupotomy to treat heel pain. (3) The control group adopted other treatment methods, including external application of plaster, acupuncture, simple acupuncture and TCM fumigation, oral Chinese and Western medicine, and other treatment methods. (4) The language of the documents was not limited.

#### 2.3.2. Diagnostic criteria

The literature included in this study met the requirements for RCTs, including age, course of disease, sex, and source of cases. Additionally, the symptoms mentioned in the literature aligned with the corresponding diagnostic criteria. The diagnostic criteria for heel pain can be found in the fourth edition of ``Practical Orthopedics’’, published by the People’s Military Medical Publishing House.^[12]^ According to these criteria, heel pain is characterized by an aseptic inflammation of the sole of the foot. Typically, there is no history of foot trauma; however, there may be a history of long-term foot fatigue, such as plantar fasciitis. The symptoms usually involve severe discomfort when the soles of the feet touch the ground in the morning, which can be relieved by appropriate activities. However, if the activity time is too long or too far, the symptoms may worsen. The affected areas are primarily affected by the load on the feet and are often located in the support area. In contrast, patients with calcaneal bursitis experience swelling and pain on the sole of the foot and may even experience fluid tremors in the affected area. Pain in these patients is usually localized primarily under the sole of the foot and the retrocalcaneal nodules.

#### 2.3.3. Intervention measures

##### 2.3.3.1. Treatment group

The treatment group underwent intervention measures, primarily utilizing TCM fumigation and acupotomy therapy. Acupotomy involves techniques such as calcaneal decompression and fascial loosening. Additionally, detoxification and other methods have been employed using acupotomy treatment techniques, along with local sealing treatment using different drugs in combination with acupotomy treatment.

##### 2.3.3.2. Control group

The control group underwent diagnosis and treatment using various methods, including conventional treatment, acupuncture, pain point sealing, acupotomy alone, and TCM fumigation alone.

#### 2.3.4. Outcome index

##### 2.3.4.1. Primary outcome measure

Efficacy, marked impact, and improvement were all considered effective treatments and indicators. The main outcome measures used were clinical effectiveness rate, cure rate, and other effective indicators.

##### 2.3.4.2. Secondary outcome measure

Secondary outcome indicators such as the visual analogue scale score, incidence of adverse events, safety, quality of life evaluation, and follow-up were utilized.

### 2.4. Document exclusion criteria

There were no studies for the control group. (1) The intervention measures of the treatment group were non-Chinese medicine fumigation combined with acupotomy therapy; there were no studies with this treatment; (2) semi-randomized, non-randomized controlled trial literature was used in the grouping of clinical observations, including grouping by order of visit time, order of visits, and odd and even number grouping by inpatient number or outpatient number. (3) All clinical observations that were not RCT studies, including abstract-only and review literature, animal experiment research, genetic and immune literature, expert clinical academic research, problem theory discussion, and typical case analysis. (4) Literature with data parameters repeatedly published; including patients with heel pain caused by fractures and dislocations, muscle injuries, osteomyelitis, bone tuberculosis, bone tumors, and other heel surgeries. (5) Unavailable full texts and inconsistencies in data and other unusable literature.

### 2.5. Literature screening and data extraction

Two researchers screened documents that met the previously established inclusion and exclusion criteria. They excluded documents that did not meet these criteria and recorded the reasons for their exclusion. The results of the included studies were crosschecked by the 2 researchers. In cases where the data in the literature were incomplete, the authors were contacted for further communication. If relevant data and information could not be obtained, the study was eliminated. Subsequently, a database was created to collect documents independently extracted by the 2 researchers. In cases in which it was difficult to determine whether to include certain documents, disagreements were resolved through discussion or with the assistance of a third evaluator. The specific content extracted included the name of the author, year of publication, age of the included patients, disease course, trial plan, implementation methods, interventions in the treatment and control groups, treatment cycle, outcome indicators, adverse events, follow-ups, and personnel who left the study midway. All data are presented in tabular form.^[13,14]^

### 2.6. Literature quality evaluation

The quality of the included studies was assessed using the random risk-of-bias assessment tool recommended by the PRISMA checklist Collaboration. The assessment tool consists of 7 items, including the method of generating random sequences, allocation concealment scheme, blinding of subjects and intervention providers, blinding of outcome evaluators, integrity of outcome data, selective reporting of outcomes, and the presence of other sources of bias. Each project was evaluated as having low, high, or unknown risk for each of these items. The evaluation process involved 2 independent researchers who assessed the studies separately and crosschecked the results. In case of any disagreement, the 2 researchers resolved the issues through discussion or with the assistance of a third research evaluator.^[13,14]^

### 2.7. Data processing and statistical analysis

Meta-analysis of the literature data involved the use of Review Manager 5.4 software to combine and analyze homogeneous data extracted from the clinical trial data for unified processing. The combined statistics represented the weighted average of the effect sizes from multiple data sources. Each research result was independently evaluated for effect size and 95% confidence interval (CI). Based on the research type and heterogeneity test data, an appropriate statistical analysis model was selected, such as the finite element method (FEM) or random-effects model. The results, represented by the 95% CI, were combined into the odds ratio (OR) or relative risk. If the 95% CI included 1, it was considered not meaningful, and if none of the 95% CI contained 1, it was considered meaningful. Heterogeneity among studies was assessed using the *Q* test and *I*². A *P*-value 50% between the studies, data pooling was not required. A sensitivity analysis was performed by systematically excluding each included study to assess the robustness of the combined results. An inverted funnel plot was used to detect potential publication bias.^[15]^

### 2.8. Evidence quality grading

The quality of evidence for the outcome indicators was graded using the internationally accepted GRADE system. The system categorizes evidence into 4 levels: high, moderate, low, and very low. All studies included in this analysis were RCTs that were considered to have the highest level of evidence. Five factors can reduce the quality of evidence: study limitations, publication bias, research imprecision, research inconsistency, and indirectness of research results. However, 3 factors can improve the quality of evidence: a large effect size, large number of responses, and addressing all possible remaining confounding factors.^[16]^

## 3. Results

### 3.1. Literature search results

**Figure 1.**
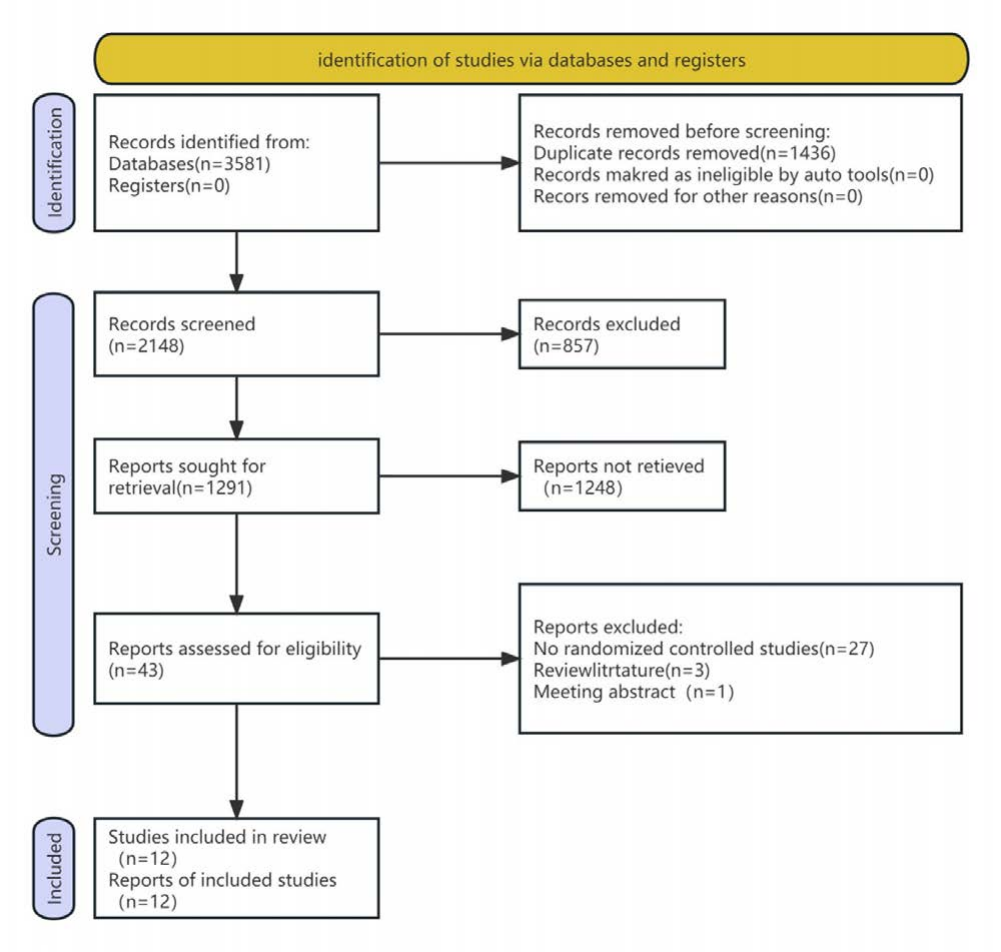
Flow chart of study identification and selection.

A total of 3581 relevant studies were initially searched, and after several rounds of checks, 12 Chinese studies were selected.^[17–28]^ All of these were clinical observational studies conducted in China. Among the 12 selected studies, the sample sizes ranged from 57 to 150, with a total of 1095 cases. The treatment and control groups consisted of 548 and 547 patients, respectively. The literature identification and selection processes are shown in Fig. 1.

### 3.2. Basic features of the data included in the study

The characteristics of the included trials are shown in Tables 1 and 2.

**Table 1:** Basic characteristics of the included literature.

Author, year	Sample (TG/CG)	Average age/yr (TG/CG)	Mean course of disease/m (TG/CG)	Intervention measure		Course of treatment	Outcome index			
				Treatment group	Control group		Total effective rate	Follow-up visit	VAS score	Security
Xue^[17]^	50/50	53.3 ± 1.8/52.6 ± 2.3	6.8 ± 1.1/6.2 ± 0.8	Acupotomy combined with TCM fumigation treatment	TCM fumigation treatment	30 days	Clinical effective rate	NR	NR	NR
Wang and Lou^[18]^	79/79	61.8 ± 4.3/62.1 ± 4.7	11.3 ± 3.2/10.8 ± 2.4	Acupotomy combined with TCM fumigation treatment	Acupotomy combined with warm water fumigation	9days	VAS score	4 to 20 weeks	Exist	Exist
Wang et al^[19]^	29/28	66.7/71.0	10.7/11.3	Acupotomy combined with TCM fumigation treatment	Acupotomy	14 days	Clinical effective rate	1 year	Exist	NR
Li^[20]^	75/75	53.9 ± 4.5/54.6 ± 4.8	5.8 ± 1.2/5.6 ± 1.1	Acupotomy combined with TCM fumigation treatment	Acupotomy	14 days	Clinical effective rate	2 year	NR	NR
Wang et al^[21]^	30/30	47.7 ± 2.2/46.2 ± 2.2	46.3 ± 6.7	Acupotomy combined with TCM fumigation treatment	Acupotomy	8 days	Clinical effective rate	1 year	NR	Exist
Zhou and Dong^[22]^	35/35	53.4/55.7	5.7/6.9	Acupotomy combined with TCM fumigation treatment	TCM fumigation treatment	28 days	Clinical effective rate	NR	NR	NR
He^[23]^	50/50	60.2/28.4	3.9/4.6	Acupotomy combined with TCM fumigation treatment	Local block therapy	20 days	Clinical effective rate	NR	NR	NR
Wang^[24]^	60/60	48.5	-	Acupotomy combined with TCM fumigation treatment	Acupotomy	21 days	Clinical effective rate	NR	NR	NR
Yin^[25]^	40/40	60.8/59.6	4.7/5.2	Acupotomy combined with TCM fumigation treatment	Local block therapy	14 days	Clinical effective rate	NR	NR	NR
Shi^[26]^	30/30	44.6	-	Acupotomy combined with TCM fumigation treatment	Acupuncture combined with TCM fumigation treatment	30 days	Clinical effective rate	NR	Exist	NR
Ou^[27]^	30/30	60.8/59.6	4.7/5.2	Acupotomy combined with TCM fumigation treatment	Local block therapy	30 days	Clinical effective rate	NR	NR	NR
Zhi^[28]^	40/40	57	–	Acupotomy combined with TCM fumigation treatment	Acupotomy	20 days	Clinical effective rate	Half a year	NR	NR

CG = control group, NR = not rated, TCM = traditional Chinese medicine, TG = treatment group, VAS = visual analogue score.

**Table 2:** Intervention measure in the meta-analysis.

Author, year	Intervention measure		
	Treatment group		Control group
	Acupotomy operation method	Chinese medicine fumigation treatment prescription	
Xue^[17]^	The acupotomy incision is performed parallel to the long axis of the foot, with the knife body angled at 60° to the skin until it reaches the calcaneal surface. The incision is repeated 1 to 2 times, followed by longitudinal dredging and transverse peeling. Next, the needle knife is elevated to the superficial surface of the aponeurosis, where the knife edge is rotated 90° before making 2 to 4 additional incisions.	20 g each of *Ligusticum chuanxiong*, *Smilax cocos*, *Clematis clematis*, *Scutellaria sibiricum*, *Scutellaria sibiricum*, Ground bark, Dandelion, Sichuan pepper, *Fructus sapiens*, Safflower and Pittosporum bark.	TCM fumigation treatment alone, the prescription was composed of the same treatment group.
Wang and Lou^[18]^	The acupotomy incision is 45° to the longitudinal axis of the foot, and the knife is inserted along the fiber direction of the plantar aponeurosis, with the tip of the knife directed toward the body of the calcaneus until it reaches the junction of the calcaneus and the plantar aponeurosis. Make the blade of the knife perpendicular to the plantar aponeurosis, and peel it horizontally at the junction of the bone and fascia 2 to 5 times. After there is a feeling of movement under the knife, turn the knife edge and release it longitudinally in the fascia body 2 to 3 times.	Eucommia 30 g, *Achyranthes bidentata* 30 g, *Rehmannia glutinosa* 18 g, Aconite 12 g, Pleurotus 15 g, Dendrobium 18 g, Dipsacus Dipsacus 18 g, Ligusticum Chuanxiong 30 g, *Angelica sinensis* 30 g	The acupotomy treatment operation method is the same as that of the treatment group, and it is combined with applying warm water to the affected foot every day.
Wang et al^[19]^	The angle of the acupotomy is perpendicular to the direction of the plantar fascia, and the ligaments and aponeurosis attached to the bone spur are cut and separated 3 to 5 times in total.	30 g each of Corydalis, Achyranthes, Achyranthes Radix, Clematis, and Sichuan Cortex, 20 g each of Corydalis Corydalis, Genchi, Myrrh, Frankincense, Lovage, Papaya, Mugwort Leaves, and Acanthopanax Bark, peach kernel, Ligusticum Chuanxiong, and Aconite Root, 15 g each of Cao Wu and Safflower.	Acupotomy treatment alone, the operation mode was the same as that of the treatment group.
Li^[20]^	The acupotomy is 90° to the sole of the foot, and the depth of the knife reaches directly to the calcaneal tubercle. Use cross incision and stripping method to release the patient’s heel.	30 g each of epimedium, psoralen, *Eucommia ulmoides*, *Achyranthes bidentata*, Millet Spatholobus, *Aphrodisiac cordata*, *Scutellaria sinensis*, and *Saponaria sinensis*, and 20 g each of Frankincense, Clematis, Millennium Jian, and Lithospermum.	Acupotomy treatment alone, the operation mode was the same as that of the treatment group.
Wang et al^[21]^	The acupotomy is inserted directly into the bone surface, aligning the knife edge with the longitudinal axis of the foot. Subsequently, the patient’s affected foot is dorsiflexed excessively, putting the plantar aponeurosis or plantar long ligament under tension. In cases of pain and swelling, longitudinal cutting and peeling should be performed 2 to 3 times, followed by horizontal peeling 2 to 3 times.	Cinnamon twig 20 g, Rhubarb 30 g, Zedoary 15 g, hematoxylin 30 g, Nodule 20 g, Schizonepeta 20 g, Parsnip 20 g, Safflower 15 g, Holly 60 g, Cork 30 g, Duhuo 20 g, *Angelica sinensis* 20 g, Clematis 30 g	Acupotomy treatment alone, the operation mode was the same as that of the treatment group.
Zhou and Dong^[22]^	The incision of the acupotomy is perpendicular to the longitudinal axis of the foot. The needle body is inserted at an angle of 60° to 80° to the plane of the heel base, reaching as deep as the tip of the bone spur or the periosteum of the calcaneus base. A horizontal incision and peeling is made 3 to 4 times or left and right, and then the knife is removed. Needle.	30 g each of *Cyperus rotunda*, red peony root, salvia miltiorrhiza, papaya, radix root, and sapsfoil, 20 g each of clematis, acanthopanax bark, and millipede, and 12 g achyranthes root.	TCM fumigation treatment alone, the prescription was composed of the same treatment group.
He^[23]^	The acupotomy incision is at an angle of 60° to 70° to the skin level, and the needle is inserted in layers, directly to the bone surface, with 2 to 5 transverse penetrations and 1 to 3 longitudinal dredging times.	Chuanxiong, Caowu, Guizhi peach kernel, safflower, Qianghuo, clematis, Shenjincao, Shujincao	Prednisolone 25 mg plus 1% lidocaine 4 to 6 mL is used for local sealing treatment on the pain point.
Wang^[24]^	The acupotomy incision is parallel to the tendon, and the needle body is perpendicular to the sole of the foot. When the needle body reaches the tenderness of the calcaneal tubercle, peel off the fibers and aponeurosis laterally, and peel it off 3 to 4 times.	30 g each of Shenjincao, Tougucao, Qianqianjian, and *Achyranthes bidentata*, 15 g each of Sichuan Wuxi and Cao Wu, 20 g each of frankincense, myrrh, safflower, asarum, Chuanxiong, saposhnikovia, and papaya.	Acupotomy treatment alone, the operation mode was the same as that of the treatment group.
Yin^[25]^	The acupotomy incision is perpendicular to the longitudinal axis of the foot. The needle body is at an angle of 60° to 80° to the plane of the heel bottom, reaching as deep as the tip of the bone spur or the periosteum of the calcaneus base. A transverse incision and peeling is performed 3 to 4 times.	20 g each of cinnamon twigs and moxa leaves, 15 g each of *Achyranthes bidentata*, aconite, *Achyranthes sinensis*, *Herba sibirica*, and *Sophora solani*, 10 g each of *Ligusticum chuanxiong* and safflower, 5 g of cloves and fennel.	Prednisolone 25 mg plus 2% lidocaine 2 ml was used to perform local sealing treatment on the painful point.
Shi^[26]^	The acupotomy incision is made perpendicularly to the long axis of the foot, and the acupuncture can only be performed when it reaches the bone surface. First make 2 to 3 incisions, remove them horizontally in a small amount, and peel them vertically in a larger amount until there is a loose feeling under the needle. Then turn the needle knife and make incisions 1 to 2 times.	20g each of Pittosporum bark, safflower, saposhnikovia, Sichuan pepper, male lettuce, ground bark, clematis, clematis, clematis, smilax, and peony root	Acupuncture treatment involves the use of flat tonic and flattening techniques, targeting specific acupuncture points such as Yanglingquan, Yinlingquan, Sanyinjiao, Chengshan, Kunlun, Taixi, and Ashi. Additionally, TCM fumigation treatment is incorporated, with the prescription composition matching that of the treatment group.
Ou^[27]^	Theacupotomye incision is perpendicular to the longitudinal axis of the foot. The needle body is at an angle of 60° to 80° to the plane of the heel bottom, reaching as deep as the tip of the bone spur or the periosteum of the calcaneus base. Make a horizontal incision and peel 3 to 4 times or shovel the needle left and right before removing the needle., cover the pinhole well, use one hand to excessively dorsiflex the affected foot, and at the same time push the thumb of the other hand toward the dorsum of the foot against the tense plantar aponeurosis and plantar long ligament of the arch, repeat this 2 to 3 times	20 g each of cinnamon twigs and moxa leaves, 15 g each of *Achyranthes bidentata*, aconite, *Achyranthes sinensis*, *Herba sibirica*, and *Sophora solani*, 10 g each of *Ligusticum chuanxiong* and safflower, 5 g of cloves and fennel.	Prednisolone 25 mg plus 2% lidocaine 2 mL was used to perform local sealing treatment on the painful point.
Zhi^[28]^	The acupotomy incision is parallel to the tendon, the needle body is at an angle of 60° to 80° to the sole of the foot, and the needle body is at an angle of 60° to 80° to the surface of the heel, reaching the tip of the bone spur. A transverse incision is made and peeled off 3 to 4 times.	20 g each of papaya, *Ligusticum chuanxiong*, Achyranthes root, saposhnikovia, salvia miltiorrhiza, Dipsacus root, 30g each of clematis, clematis, Acanthopanax bark, Milletia sinensis, sappan wood, and adenophora.	Acupotomy treatment alone, the operation mode was the same as that of the treatment group.

TCM = traditional Chinese medicine.

### 3.3. Quality evaluation of included studies

**Figure 2.**
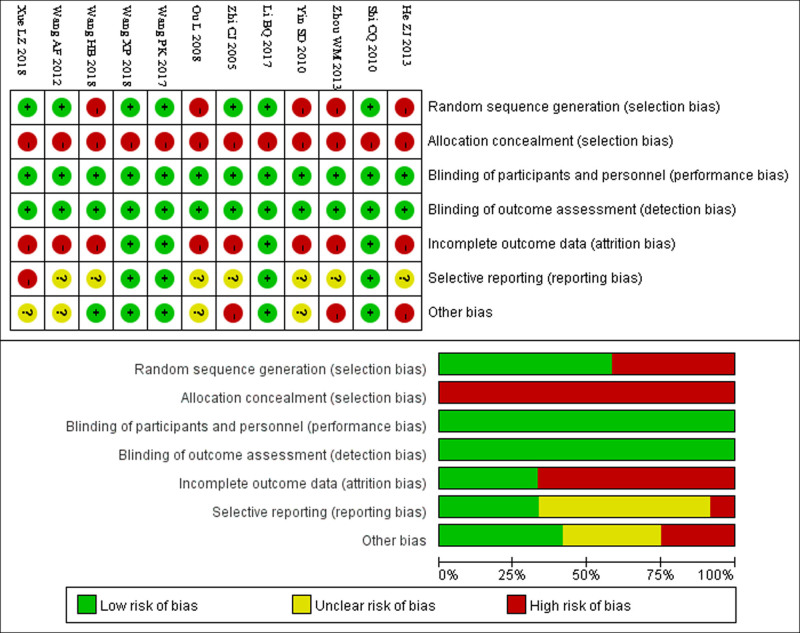
Quality evaluation map of the included literature.

The 12 included studies used random allocation models. Allocation concealment, blinding, selective reporting of results, and other biases were evaluated within the reference ranges of the quality assessment of the literature. Data were recorded and compared. The PRISMA checklist risk-of-bias assessment tool was used to systematically evaluate the quality of the 12 studies. Among the 12 included studies, all achieved low risk of bias in blinding the research subjects and interventionists, as well as blinding the outcome evaluators. However, all studies were rated as high risk of bias in terms of allocation concealment of the randomization scheme. In terms of random order generation, 7 studies had low risk of bias while 5 studies had high risk of bias. Regarding completeness of outcome indicator data, 4 studies had low risk of bias while the remaining 8 studies had high risk of bias. Concerning the possibility of selective reporting of results, 4 studies had low risk of bias, 7 studies had unclear bias, and 1 study had high risk of bias. In terms of other aspects of bias, 5 studies had low risk of bias, 4 studies had unclear bias, and 3 studies had high risk of bias. The review indicates varying quality among the included studies. Notably, 3 studies Li BQ (2017), Wang PK (2017), and Wang XP (2018), were deemed of higher quality compared to the rest. Further details are shown in Fig. 2.

### 3.4. Meta-analysis of clinical efficacy of TCM fumigation combined with acupotomy in the treatment of heel pain

**Figure 3.**
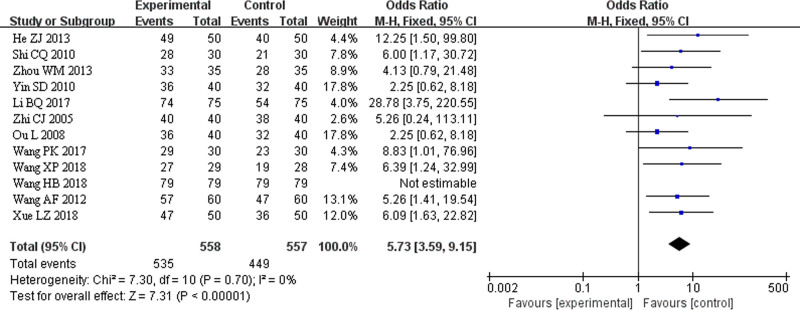
The total efficient forest map was systematically evaluated.

Twelve studies involving 1095 patients (548 in the treatment group and 547 in the control group) reported clinical efficacy. The heterogeneity test indicated no statistical heterogeneity among the 12 studies (*I*² = 0%). Therefore, FEM was used for the combined effect size analysis. The results of the meta-analysis revealed a statistically significant clinical difference between the treatment and control groups (*Z*-value = 7.31, *P* < .00001). The combined effect sizes and 95% CI for clinical efficacy were OR = 5.73, 95% CI (3.59, 9.15). This suggests that TCM fumigation combined with acupotomy has a more significant clinical curative effect in the treatment of heel pain than in the control group (Fig. 3).

### 3.5. Sensitivity analysis

Among the 12 included studies, one was removed at the time of screening, leaving 11 studies for reconducting the meta-analysis. The homogeneity was satisfactory; thus, a fixed-effects model was employed for the analysis. After excluding the literature individually, the heterogeneity and effect scales were analyzed, and the obtained results were essentially consistent with those before exclusion. This indicates that the findings of the study are reliable.^[29]^

### 3.6. Adverse reactions

Among the included studies, Wang PK (2007) reported only one case of rash and itching that resolved after treatment. Wang HB (2018) selected 158 cases and found no instances of blood vessel injury, nerve injury, or pinhole infection. None of the other literature sources mentioned any adverse reactions and none of the patients experienced local skin redness, itching, or herpes. When symptoms occurred, the patients were effectively treated and their condition improved.

### 3.7. Publication bias assessment

**Figure 4.**
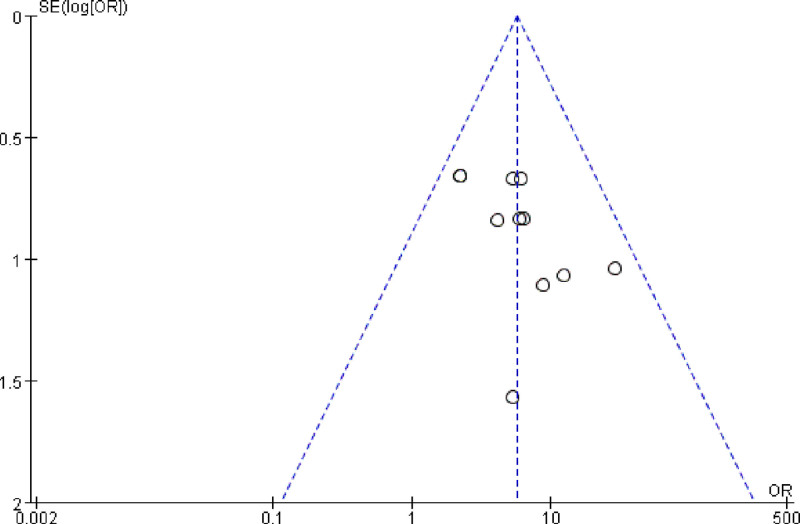
The funnel plot of total effective rate was evaluated.

Publication bias was assessed in the included studies (Fig. 4). The funnel plot shows asymmetry, indicating publication bias.

### 3.8. Subgroup analysis

#### 3.8.1. Territory

**Figure 5.**
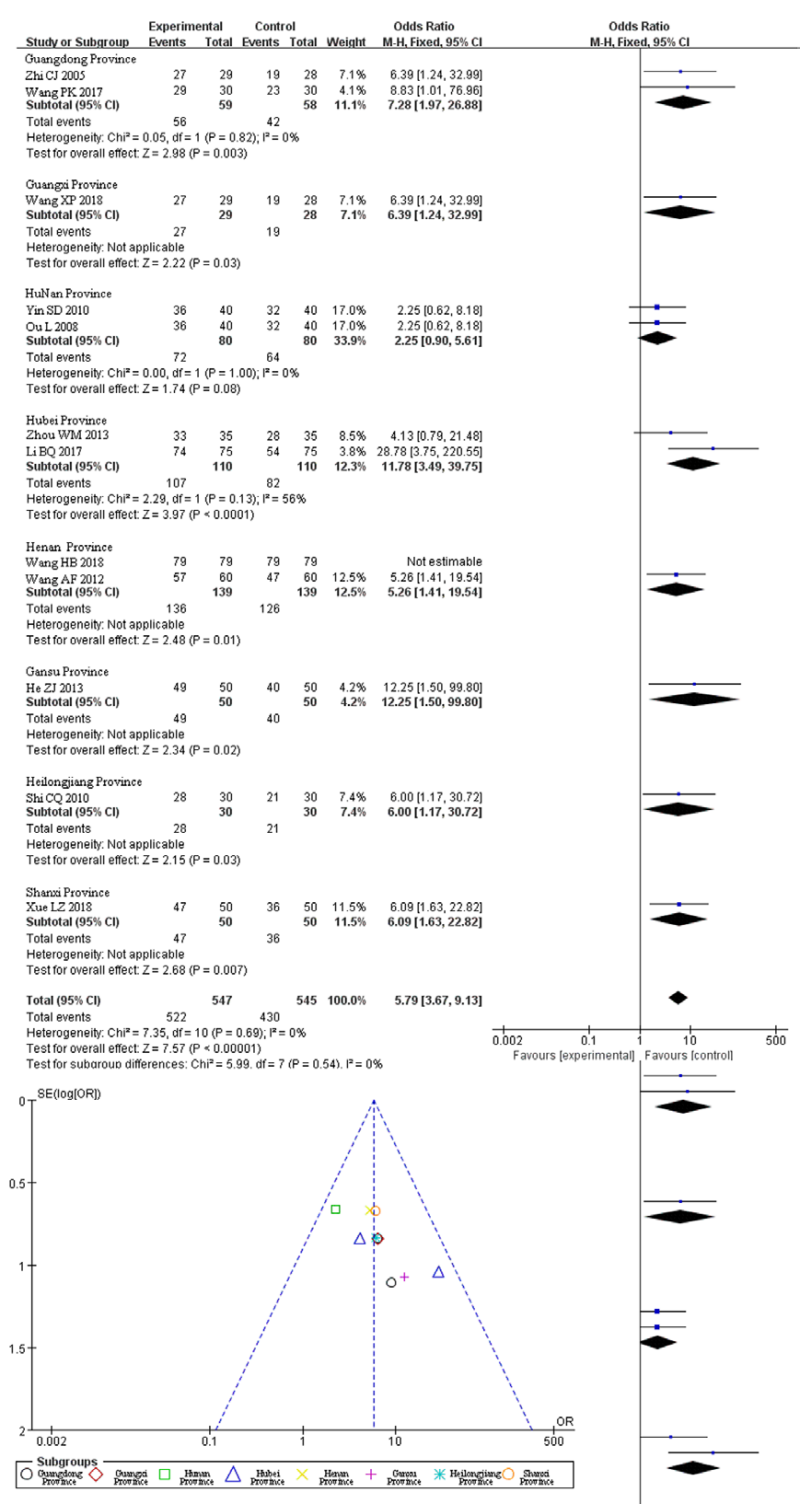
Total effective forest map and funnel map in different regions.

This systematic review examined the effects of TCM fumigation combined with acupotomy on heel pain in different provinces of China. This review includes 8 provinces: Hubei, Hunan, Henan, Guangdong, Guangxi, Gansu, Heilongjiang, and Shanxi. Considering the variations in living customs, climate, and economic levels among these provinces, people’s quality of life differs. This study combined outcome indicators from each province to observe regional differences in therapeutic effects. The forest plot of the total effective rate in different regions showed low heterogeneity, indicating that the population of different provinces did not significantly affect the heterogeneity (*I*² = 0%). The meta-analysis revealed a statistically significant clinical difference between the treatment and control groups, with a *Z*-value of 7.57 (*P* < .00001). The combined effect size and 95% CI for clinical efficacy were OR = 5.79, 95% CI (3.67, 9.13). Notably, Gansu Province has the highest OR value of 12.25, 95% CI (1.50, 99.80), followed by Hubei Province with an OR value of 11.75, 95% CI (3.49, 39.75). These findings suggest that washing combined with acupotomy is more effective for treating heel pain. However, the number of literature sources and samples included from each province was relatively small, with some provinces having only one literature source on the clinical observation of TCM fumigation combined with acupotomy treatment for heel pain. Therefore, the research conclusion should be interpreted with caution and further confirmed by large-sample, high-quality clinical trials.^[30]^ An assessment of publication bias in the included studies was conducted, and the funnel plot exhibited asymmetry, indicating the presence of a publication bias (Fig. 5).

#### 3.8.2. Intervention measures

##### 3.8.2.1. Meta-analysis of TCM fumigation combined with acupotomy compared with acupotomy alone

**Figure 6.**
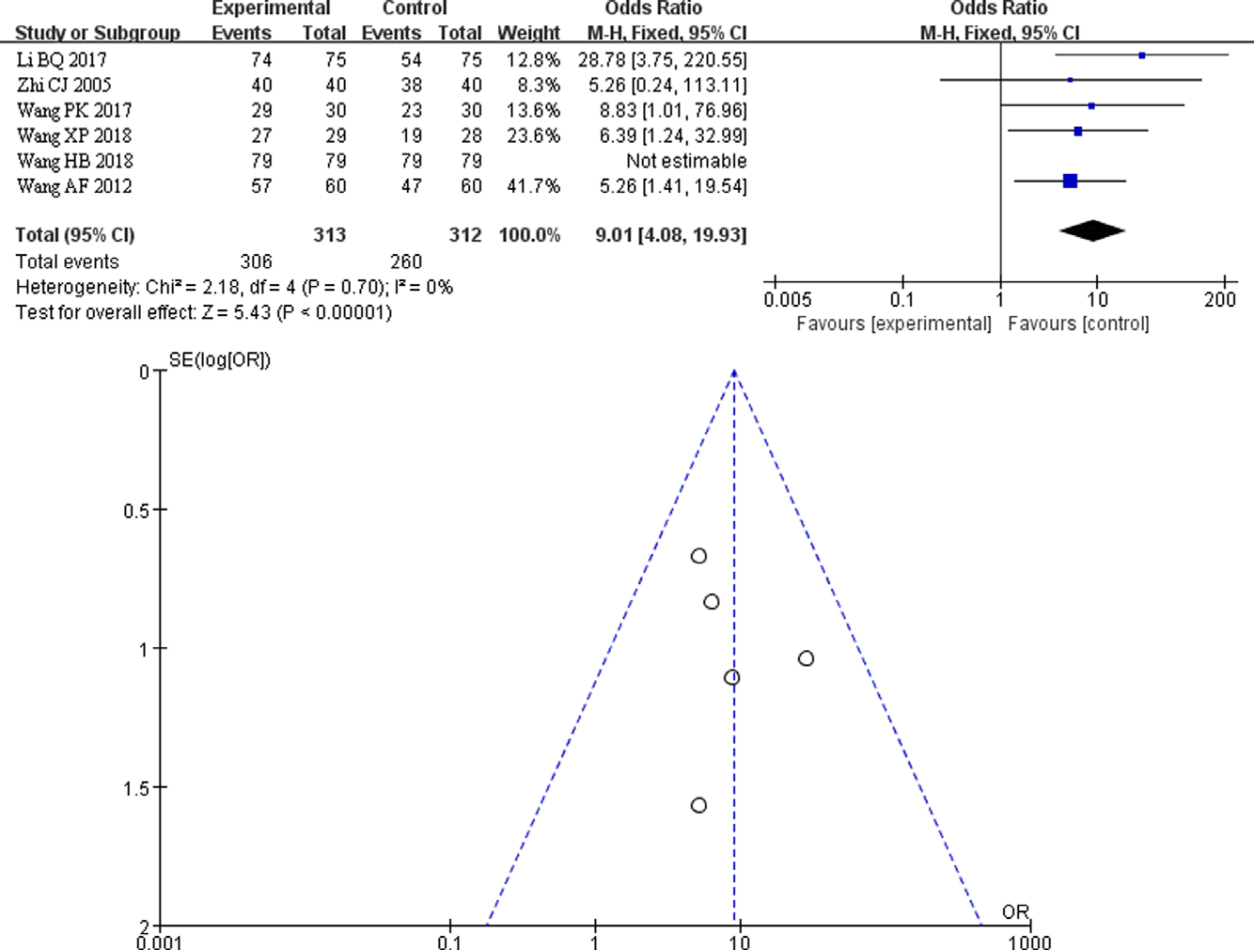
Forest map of TCM fumigation combined with acupotomy compared with acupotomy alone. TCM = traditional Chinese medicine.

In the control group, 6 studies used acupotomy alone These studies include Li BQ (2017), Wang PK (2017), Wang AF (2012), Zhi CJ (2005), Wang XP (2018), and Wang HB (2018). Notably, Wang HB (2018) included acupotomy combined with soaking feet in warm water-in the control group. The warm water used in this study did not contain any medicines. However, it is important to mention that there is no evidence indicating whether patients in the control group in other studies also soaked their feet in warm water. Therefore, for the purpose of this study, the literature was merged with other studies. In total, the 6 studies included 625 patients, with 313 cases in the treatment group and 312 cases in the control group. The heterogeneity test revealed no statistical heterogeneity among the 6 studies (*I*² = 0%). Consequently, FEM combined with effect size analysis was employed. The results of the meta-analysis demonstrated a statistically significant clinical difference between the treatment and control groups, with a *Z*-value of 5.43 (*P* < .00001). The combined effect size and 95% CI of clinical efficacy were calculated as OR = 9.01, 95% CI (4.08, 19.93). This indicates that TCM fumigation and acupotomy had a more significant clinical curative effect on heel pain than in the control group. Publication bias was assessed in the included studies, and the funnel plot exhibited asymmetry, indicating the presence of publication bias (Fig. 6).

##### 3.8.2.2. Meta-analysis of TCM fumigation combined with acupotomy therapy compared with simple blocking therapy

**Figure 7.**
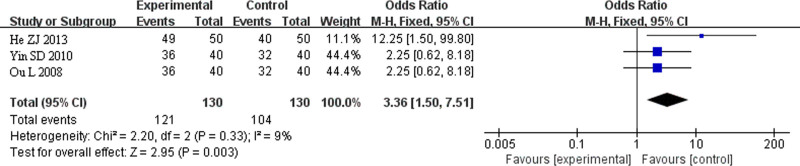
Forest map of TCM fumigation combined with acupotomy compared with local block therapy alone. TCM = traditional Chinese medicine.

In the control group, 3 studies used simple closed therapy: He ZJ (2013), Yin SD (2010), and Ou L (2008). These studies included 260 patients, with 130 cases in the treatment group and 130 in the control group. The heterogeneity test indicated no statistical heterogeneity among the 3 studies (*I*² = 0%). Therefore, FEM was used for the combined effect size analysis. The results of the meta-analysis showed a statistically significant clinical difference between the treatment and control groups, with a *Z*-value of 2.95 (*P* < .00001). The combined effect size and 95% CI for the clinical efficacy were OR = 3.36, 95% CI (1.50, 7.51). This indicates that TCM fumigation and acupotomy have a more significant clinical curative effect on heel pain than in the control group (Fig. 7). In the subgroup analysis comparing TCM fumigation combined with acupotomy to simple closed therapy, only 3 included studies were available for evaluation of publication bias; therefore, no funnel plot evaluation was performed.

##### 3.8.2.3. Meta-analysis of TCM fumigation combined with acupotomy compared with fumigation with TCM alone

**Figure 8.**
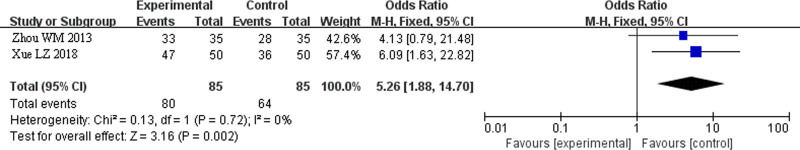
Forest map of TCM fumigation combined with acupotomy compared with TCM fumigation alone. TCM = traditional Chinese medicine.

In the control group, 2 studies, Xue LZ (2018) and Zhou WM (2013), used simple closed therapy. The total number of patients in the 2 included studies was 170, with 85 cases in the treatment group and 85 in the control group. The heterogeneity test showed no statistical heterogeneity between the 2 studies (*I*² = 0%). Therefore, FEM combined with effect size analysis was used. The results of the meta-analysis indicated a statistically significant clinical difference between the treatment and control groups, with a *Z*-value of 3.16 (*P* < .00001). The combined effect size and 95% CI of clinical efficacy were OR = 5.26, 95% CI (1.88, 14.70). This suggests that, compared to the control group, the clinical curative effect of TCM fumigation combined with acupotomy in treating heel pain is more significant (Fig. 8). In the subgroup analysis of TCM fumigation combined with acupotomy versus simple TCM fumigation, only 2 included literatures were not suitable for evaluating publication bias, therefore funnel plot evaluation was not performed.

#### 3.8.3. Combined analysis of other outcome indicators

For heel pain, various standard scoring indicators are used, such as the visual analogue score for pain, Jadad scale (modified), American Orthopedic Foot and Ankle Society ankle-hindfoot scoring scale, foot function index evaluation form, and TCM symptom score. In the 12 included studies, there were significant differences in the scoring standard outcome indicators themselves as well as in various complex factors such as the patient’s sex, age, and disease duration. Additionally, the research design schemes, acupotomy techniques, TCM fumigation and washing prescriptions, and treatment cycles varied greatly among the studies. Because of these variations, limited samples were available for this study. Consequently, it was not possible to uniformly evaluate the abovementioned indicators, leading to their exclusion from the evaluation.

### 3.9. Evidence quality grading of outcome indicators

**Figure 9.**
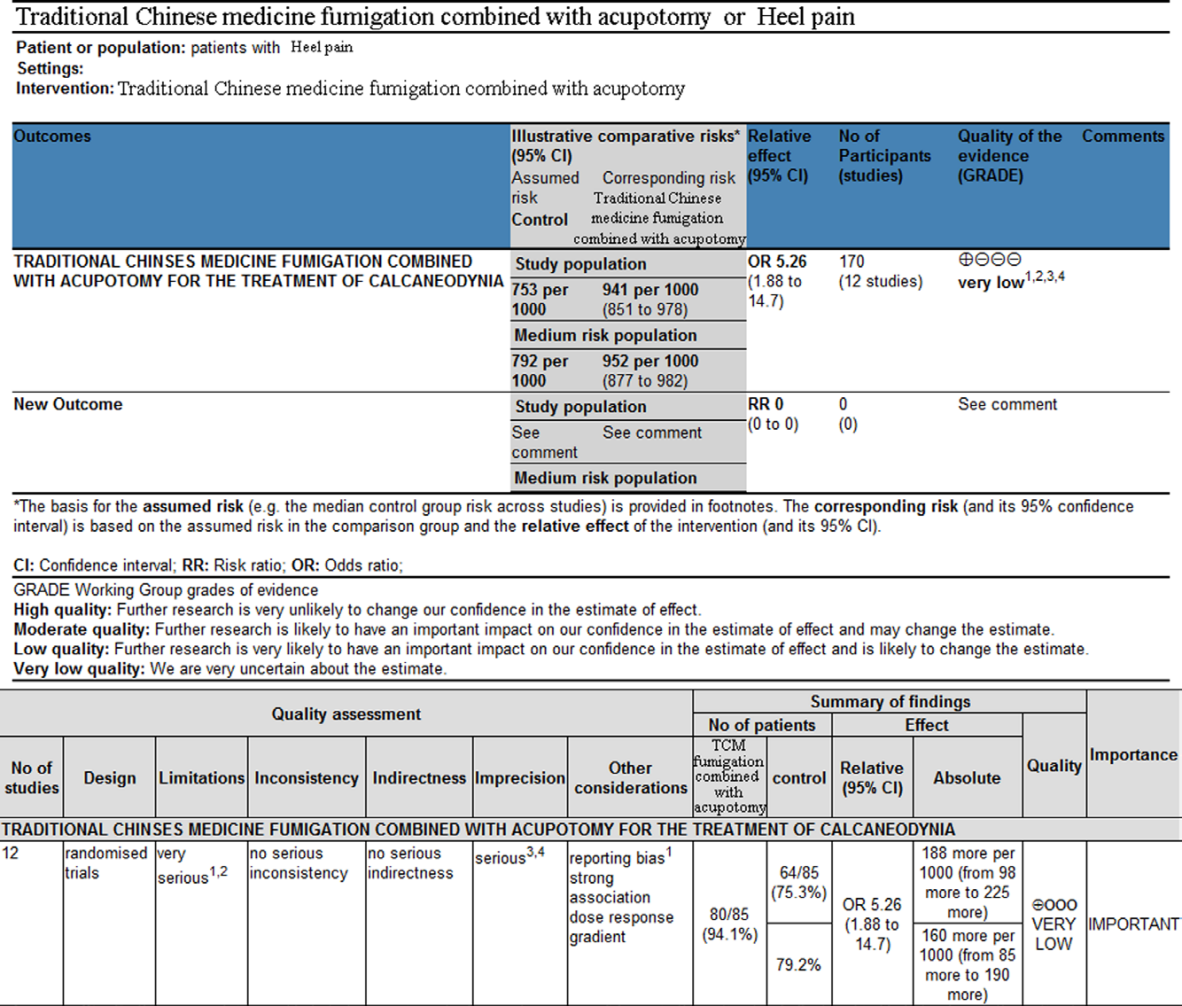
GRADE system evidence quality map. GRADE = Grading of Recommendations Assessment, Development and Evaluation.

The GRADE profiler 3.2.2 software was used to grade the evidence for the outcome indicators (Fig. 9). The level of evidence for the comparison between the TCM fumigation and acupotomy treatment group and the conventional therapy group was determined to be very low.^[31]^

## 4. Discussion

Fumigation and washing using TCM have a long history in China. The main objective is to utilize the warming effect of the medicinal liquid to dilate blood vessels and increase blood flow. This promotes metabolism and nutrient absorption in the affected area.^[32]^ Additionally, they help regulate the function of nerves and muscles, and the local absorption of herbal medicines directly affects the affected area. Although the herbal medicines studied varied among the 12 included studies, they all centered around the addition or subtraction of Pittosporum bark decoction. Haitongpi Decoction is commonly used in orthopedics and traumatology departments to treat bruises and chronic strains, particularly effective for patients with heel pain. The core component of Pittosporum bark decoction is Pittosporum bark, known for its ability to remove rheumatism and unblock meridians. Acanthopanax bark, Tougucao, Shenjincao, Clematis, Yuanhu, safflower, and mugwort leaves work together to dispel wind, unblock meridians, relieve pain, and warm the body. Dandelion and Viola purpurea are heat-clearing and detoxifying, enhancing their ability to eliminate carbuncles and reduce swelling. *Sophora flavescens* promotes blood circulation and regulates Qi, while Sichuan pepper and cinnamon dispel wind and cold, nourishing kidney yang. Asarum acts as an inducing drug for the kidney meridian. Overall, this prescription effectively treats heel pain by addressing both symptoms and root causes through a combination of herbs that work synergistically to regulate the body’s circulation and nourishment. Acupotomy therapy is a noninvasive method that can effectively alleviate heel adhesions, scars, and contractures. It also improves the metabolism of the surrounding tissues, enhances local venous return, and accelerates the absorption of inflammation. However, it cannot address all symptoms of plantar pain.^[33–35]^ Clinical studies have shown that combining fumigation and washing of TCM with acupotomy treatment for heel pain can provide the benefits of both TCM and Western medicine. This combination not only clears meridians and improves blood circulation but also strengthens capillaries on the soles of the feet. By accelerating blood supply and improving microcirculation, it promotes tissue repair, softens bone spurs, and facilitates rapid absorption of inflammation. Ultimately, this integrated approach helps to restore the dynamic balance of local tissues and effectively relieves heel pain symptoms, aligning with the green treatment trend.

Throughout history, TCM has relied on the patient’s subjective experience and the doctor’s physical examination as the standard for evaluating pain. However, it lacks standardized, objective, and quantifiable evaluation criteria. The advent of evidence-based medicine has brought gradual advancements in disease observation and research, providing a scientific basis for the systematic evaluation of clinical treatments. Meta-analyses play a crucial role in consolidating and analyzing the literature in evidence-based medicine. It saves time and resources, harmonizes research from multiple sources, expands the sample size, and yields more meaningful information, thereby enhancing the efficiency of the resulting analysis. Moreover, this study provided an objective foundation for guiding clinical diagnosis and treatment.

The GRADE evidence grading system, recognized by numerous international organizations and institutions worldwide, evaluates the overall evidence of a group of literature rather than individual studies, making the resulting outcome measures more credible, powerful, and objective.^[36,37]^ This evaluation employs an evidence-based medicine approach to assess the included studies comprehensively. The GRADE evidence classification was used to determine the quality of the included literature to ensure a reliable evaluation free from subjective human factors. Evidence for the effectiveness of TCM fumigation combined with acupotomy treatment in pain management was graded as VERY LOW. It is important to note that the included studies consisted mostly of Chinese sources, which may introduce a certain bias owing to the generally lower quality of these publications. In addition, the literature lacks consistency in terms of acupotomy therapy duration, herbal medicine prescriptions, and acupotomy operation methods. The inclusion and evaluation of literature could not completely eliminate subjective and human factors. Moreover, only 5 of the 12 selected documents conducted patient follow-up, and a significant number of documents did not provide posttreatment follow-up. Furthermore, only 2 articles included a safety evaluation, which hindered the establishment of convincing evidence regarding the safety of TCM fumigation combined with acupotomy in the treatment of heel pain. It is important to acknowledge that the objectivity of the results may still be subject to change after consultation with a third evaluator. In the future, high-quality literature may be added to the evaluation system, potentially leading to different conclusions. Therefore, caution should be exercised while referring to the conclusions of this systematic review.

## 5. Limitations of the current study

Through a systematic review, the content of the literature in the electronic databases and the scope of manual retrieval were limited. The retrieved content consisted mainly of Chinese journal articles, with very few international journal articles. This limitation may have resulted in some relevant studies not being included. Additionally, the included studies were insufficiently comprehensive. Many of the retrieved articles were single case reports without a control group experiment, making the findings unreliable and unsuitable for adoption. A systematic review revealed no universally accepted standard for the diagnosis of heel pain. Instead, diagnostic criteria were derived from various sources, such as literature, books, and association guidelines available on the Internet, leading to a lack of uniformity among the selected patient cases. Furthermore, there is no standardized and accurate scoring system for assessing the severity of heel pain. Commonly used scoring standards include the American Orthopedic Foot and Ankel Society ankle-hand-foot scale, foot function index evaluation form, simplified McGill pain scale questionnaire, and modified Jadad scale. Although these scoring systems have been widely used in clinical practice and serve as valuable medical references, they lack laboratory tests and imaging evaluations for quantitative assessments before and after treatment. The absence of standardized quantification and objectivity in these evaluations is a limitation as individual pain thresholds and sensitivities vary, which can affect the assessment of treatment effectiveness.^[38–41]^ This systematic review includes only published literature that reported positive results. There may be unpublished literature or literature related to the confidentiality of the topic with negative research results; however, these were not included in the review, potentially leading to biased results.

## 6. Conclusions

In conclusion, we conducted a systematic review and meta-analysis using the GRADE system to assess the effectiveness of TCM fumigation and acupotomy in the treatment of heel pain. Our findings suggest that this treatment approach is more effective than a simple drug application. However, the number of studies included in our analysis was limited. Therefore, further research with larger sample sizes and longer follow-up periods is necessary to establish definitive conclusions regarding the efficacy of TCM fumigation and acupotomy in reducing heel pain.

## Acknowledgments

Thanks to the Natural Science Foundation of Heilongjiang Province surface fund for funding this research. We would like to thank Editage (www.editage.cn) for English language editing.

## Author contributions

**Conceptualization:** Chen Li, Zhi-Wen Sun, Xi Gao.

**Data curation:** Zhi-Wen Sun, Cheng-Yuan Li.

**Formal analysis:** Chen Li, Zhi-Wen Sun, Jia-Xin Ma, Cheng-Yuan Li.

**Funding acquisition:** Xi Gao.

**Investigation:** Chen Li, Zhi-Wen Sun, Jia-Xin Ma.

**Methodology:** Chen Li, Zhi-Wen Sun, Xi Gao, Hong-Jun Lou.

**Project administration:** Xi Gao, Hong-Jun Lou.

**Resources:** Chen Li, Zhi-Wen Sun, Xi Gao.

**Software:** Chen Li, Cheng-Yuan Li.

**Supervision:** Xi Gao, Hong-Jun Lou.

**Validation:** Xi Gao, Hong-Jun Lou.

**Visualization:** Chen Li, Zhi-Wen Sun.

**Writing – original draft:** Chen Li, Zhi-Wen Sun, Jia-Xin Ma.

**Writing – review & editing:** Xi Gao, Hong-Jun Lou.
